# When tyrosine kinase inhibitor sunitinib can be discontinued in metastatic renal cell carcinoma to pancreas: a case report

**DOI:** 10.1186/s13256-018-1597-z

**Published:** 2018-03-20

**Authors:** Yassir Sbitti, Adil Debbagh, Khaoula Slimani, Mohamed Mahi, Hassan Errihani, Mohamed Ichou

**Affiliations:** 1 Department of Medical Oncology, University Military Hospital of Rabat, Hay Ryad, 10000 Rabat, Morocco; 2 Department of Radiology, University Military Hospital, 10000 Rabat, Morocco; 3 Department of Medical Oncology, National Institute of Oncology, 10000 Rabat, Morocco; 4 Faculty of Medicine and Pharmacy of Fes, Sidi Mohamed Ben Abdellah University, Fez, Morocco; 5 Faculty of Medicine and Pharmacy of Rabat, Mohammed V University, Rabat, Morocco

**Keywords:** Sunitinib, Metastatic renal cell carcinoma, Treatment discontinuation, Tyrosine kinase inhibitors, Outcome

## Abstract

**Background:**

Long-term survival with durable response remains possible in the area of targeted therapies. Discontinuation of sunitinib could improve quality of life and reduce treatment costs in metastatic renal cell carcinoma with long-term disease stabilization. We discuss a case of successful interruption of antiangiogenic therapy in a patient with persisting evidence of metastases. The discontinuation of antiangiogenic therapy seems to be an option, even in indolent oligo-metastatic renal cell carcinoma with long disease stabilization before sunitinib. This observation contributes important data to the ongoing discussion on the discontinuation of treatment with kinase inhibitors in selected patients with metastatic renal cell carcinoma.

**Case presentation:**

We report a case of an 80-year-old Moroccan man treated for renal clear cell carcinoma with multiple pancreatic metastases. He was not on any other medications. He underwent active surveillance with deferred sunitinib at disease progression. He showed significant disease control on sunitinib therapy demonstrating partial response with stable disease after a total of 28 months of therapy. He experienced toxicities which were manageable with supportive care and dose adjustments. Our patient asked for a break of the sunitinib administration, and the treatment was stopped. The disease remained stable after 13 months’ discontinuation of sunitinib therapy. The patient was in excellent overall health.

**Conclusions:**

All available agents for metastatic renal cell carcinoma have side effects, which may become serious in a minority of patients. Clinicians and patients must therefore carefully balance the goals of maximal efficacy with minimal toxicity. Sunitinib can be discontinued without negatively impacting outcomes in indolent disease. Further research is needed to characterize the molecular determinants of response and resistance to targeted therapy.

## Background

New targeted therapies play a pivotal role in the modern treatment of patients with metastatic renal cell carcinoma (mRCC) and we now have several first- and second-line treatments options [1–8]. Sunitinib is an orally administered multitarget tyrosine kinase inhibitor (TKI) used as a first-line therapy for mRCC, which has demonstrated longer progression-free survival (PFS), higher response rates, and longer overall survival (OS) than interferon alfa [1]. Partial response is reported in about 31–44% of patients treated with sunitinib and complete response is reported in only about 3% of cases [1, 2]. Depending on the course and the response, the targeted therapy may last for years. The goal of treatments is still palliative. Despite their initial effectiveness in providing tumor control, targeted agents are not curative, and minorities of patients survive beyond 5 years from initiation of therapy. Moreover, all available target agents have considerable side effects that could compromise quality of life and cause economic burden to patient and society. Given the balance of toxicity and benefit with antiangiogenic-targeted therapy in patients with mRCC, discussion of discontinuation therapy is intriguing question in patients with mRCC; essentially, patients with an initial response to treatment can maintain disease control off all therapy for a period of time. Here, we report a case of sunitinib discontinuation in patient achieving stable disease after a partial remission for long duration. This observation contributes important data to the ongoing discussion on the discontinuation of treatment with kinase inhibitors.

## Case presentation

**Fig. 1:**
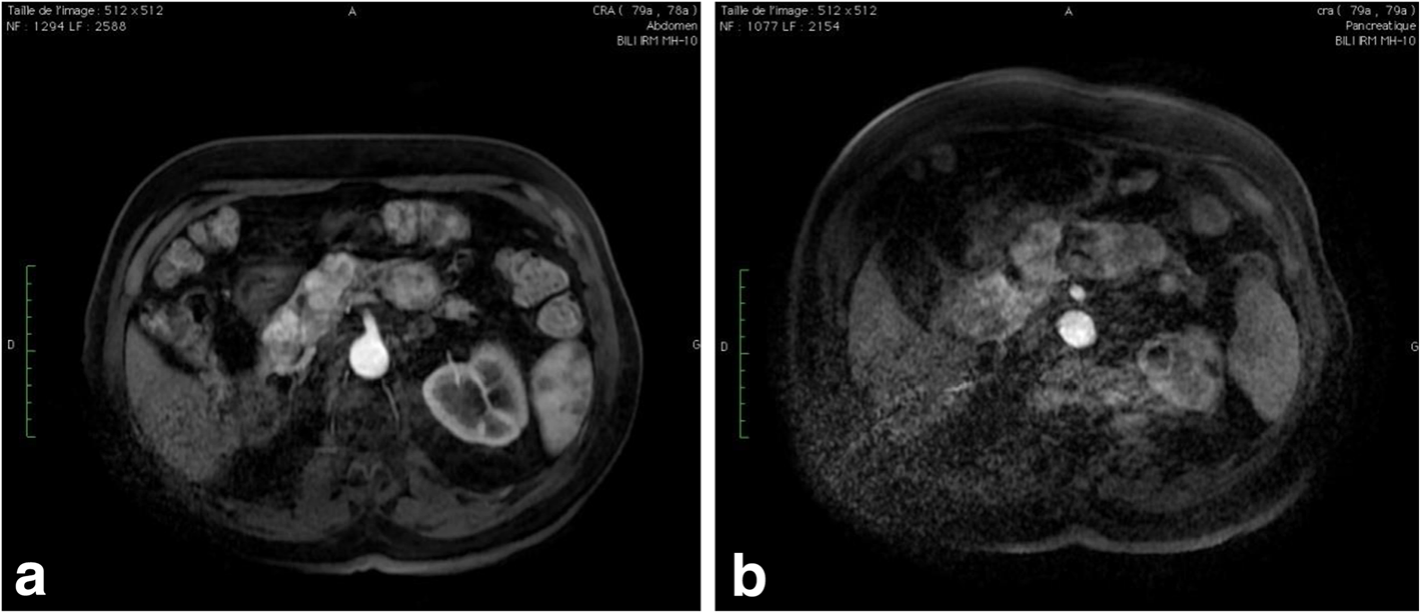
Magnetic resonance imaging shows the exemplary course of pancreatic metastases from renal cell cancer. The magnetic resonance imaging at the *left* side was performed directly before the treatment with a tyrosine kinase inhibitor was stopped in July 2016 (**a**), the MRI at the *right* side 13 months later in August 2017 (**b**).

Our patient was an 80-year-old, Moroccan man. He was an agricultural engineer. He was a nonsmoker and did not drink alcohol. There was no history of chronic diseases and chronic medications. In 1996, a right-sided nephrectomy was performed due to a localized renal cell carcinoma. Fourteen years later, in May 2010, multiples nodules in his pancreas were discovered in a follow-up abdominal computed tomography (CT) scan for which a biopsy was indicated. On admission, our patient appeared in good general condition. His temperature was 37 °C; blood pressure, 130/85 mmHg; pulse rate, 73 beats per minute; and respiration rate, 15 per minute, weight 69 kg, and height 172 cm. On physical examination, his abdomen was soft, painless, without peritoneal symptoms. His neurological system was unremarkable. His cranial nerves were intact and power in his upper and lower limbs were 5/5 throughout. A histologic examination of the biopsy revealed the diagnosis of a pancreatic metastasis from renal cell carcinoma. A complete body scan for staging was performed. Isolated metastases involving the pancreas were detected. He met International Metastatic Renal Cell Carcinoma Database (IMDC) favorable-risk criteria, with a Karnofsky performance score of 90% and corrected calcium and blood counts within the normal range, including hemoglobin (Hb) of 14.2 g/dL (normal range 13.5–17.0). Serum creatinine was 11 mg/L (estimated creatinine clearance 86 mL/min). Indolent disease based on body CT imaging with 4 years of follow-up was recognized. Active surveillance with deferred sunitinib at disease progression was performed. An abdominal CT scan demonstrated an increase in the size of his pancreatic lesions in May 2014. A complete blood count was normal (Table 1). His hepatitis B and C screenings were negative. His echocardiograph was normal for left ventricular ejection fraction and his baseline performance score was 0. We decide to start treatment with sunitinib 50 mg daily for 4 weeks of treatment followed by 2 weeks off. Evaluation of the tumor response was done according to response evaluation criteria in solid tumors by spiral CT scan, and after three cycles of sunitinib observed a partial response (30% reduction in size and 50% density of pancreatic lesions). In September 2014, our patient reported different side effects, like headache, the onset of grade III skin rash, mucosal toxicity, and gastrointestinal toxicity managed by supportive care and dose interruption. Hence the dose was reduced to 37.5 mg in a 3/4 schedule. He received 15 cycles of oral sunitinib. In November 2015, restaging CT scan after this regimen continued to show stable disease consistent with partial response per response evaluation criteria in solid tumor. He developed weakness and grade II hypothyroidism (Table 1) managed by one thyroxin supplementation. Sunitinib was reduced at dose of 25 mg (schedule 2/1). Therefore, our patient asked for a break of the sunitinib administration, and the treatment was stopped after a 9-month period. After receiving therapy for 28 months, sunitinib therapy was therefore discontinued in September 2016. Nowadays; our patient is under oncologic follow-up. He is still maintaining stable disease, excellent overall heath and a 0 performance score. Time of disease control after sunitinib discontinuation was 13 months. A follow-up abdominal magnetic resonance imaging (MRI) evaluation every 4 months revealed stable disease (Fig. 1a and b). Nowadays, the patient emphasizes feeling much better after treatment with sunitinib ended. Pancreatic metastases from renal cell cancer showed no signs of progression, neither clinically nor in MRI.

**Table 1:** Major laboratory results according to 03 cycles since sunitinib administration for 28 months

Parameter	May 2014	August 2014	November 2014	February 2015	May 2015	August 2015	November 2015	February 2016	May 2016	September 2016
Hb (g/dL)	14.2	13.3	13.5	13.7	13.2	13.6	13.9	14.1	13.8	13.9
WBC (× 10^9^/L)	4.89	4.10	5.02	4.67	4.90	5.15	4.77	4.07	5.08	4.78
PLT (× 10^9^/L)	205	198	175	189	176	154	132	120	115	120
TB (umol/L)	3	2	3	3						
ALT (U/L)	16	19	20	14	19	21	22	16	15	14
AST (U/L)	12	11	13	13	14	13	15	17	13	11
BUN (g/L	0.25	0.25	0.32	0.40	0.4	0.42	0.42	0.39	0.31	0.43
BCr (mg/L)	11	11	12	11	9	10	11	11	10	11
Proteinuria	0	0	0	0	0	0	0	0	0	0
TSH (microUI/mL)	1.34	2.09	2.98	3.34	6.02	19.16	15.68	9.45	7.45	5.69

Normal ranges are given in parentheses as follows: *Hb* hemoglobin, *WBC* white blood cell (4.0–10.0 × 10^9^/L)*, PLT* platelet (100–300 × 10^9^/L), *TB* total bilirubin (1.7–22.5 μmol/L), *ALT* alanine aminotransferase (5–45 U/L), *AST* aspartate aminotransferase (5–45 U/L), *BUN* blood urea nitrogen (0.17–0.49 g /L), BCr Blood creatinine (7–12 mg/L), *TSH* thyroid-stimulating hormone (0.27–4.20 microUI/mL)

## Discussion

Our 80-year-old patient achieved stable disease after a partial remission for long duration under sunitinib for metastatic renal cell carcinoma. After sunitinib discontinuation, progression-free survival was 13 months. Our observation contributes important data to the ongoing discussion on the discontinuation of treatment with kinase inhibitors. This case demonstrates that patients with mRCC can be taken off antiangiogenic-targeted therapy and suggests that continuous sunitinib may not be necessary in all patients. Although targeted therapies are currently a standard frontline therapy for metastatic renal cell carcinoma patients with a good or intermediate prognosis according to several phases III studies [1–12]. These therapeutic agents have substantially improved patient outcomes. Objective responses, mostly partial responses, are observed in approximately 8% to 39% of patients with median OS of more than 2 years observed with sunitinib [1, 2]. These targeted agents should be continued in cancer therapy until disease progression or toxicity, especially in the targeted therapy era [1, 13, 14]. However, in view of the fact that target agents of mRCC in general remains a palliative in the majority of cases, accompanied by chronic adverse events such as fatigue, diarrhea, hand-foot syndromes, proteinuria, and renal insufficiency, impairment of quality of life, and also the high cost of continuous long-term therapy of these therapies are becoming increasingly important issues. A key question surrounding long disease control for long periods is whether or not treatment can be discontinued without negatively impacting outcomes and whether this will reduce treatment-related side effects and improve quality of life. In routine clinical practice, 20–30% of patients experienced grade 3/4 toxicities, and treatment modifications occurred in 50–55% patients because of adverse events. Finally, up to 20% patients discontinued TKI therapy because of adverse events [1, 2, 13]. Our case illustrates achievement of partial response with sunitinib and prolonged sustained response even after sunitinib discontinuation. Several retrospective studies have suggested that discontinuation of TKI therapy is possible in carefully selected patients and may improve symptoms of toxicity [15, 16]. In contrary, preclinical models have mentioned rebound effect, with rapid regrowth and development of metastases observed after treatment discontinuation with TKIs [17]. In addition, continuation of angiogenesis inhibition in mRCC is supported by clinical evidence that switching to another vascular endothelial growth factor (VEGF) inhibitor in mRCC may increase OS in patients previously treated with sunitinib. However, this strategy had been tested in clinical practice with ambiguous results [18, 19]. In clinical practice Iacovelli and colleagues analyzed the follow-up of 63 patients with mRCC after discontinuation of anti-vascular endothelial growth factor receptor (VEGFR) TKI. They found that tumor regrowth after discontinuation of therapy was related to the reason for discontinuation; regrowth was higher in patients who discontinued treatment because of disease progression, and lower in patients who discontinued treatment because of a sustained response [20]. Koo and colleagues report that VEGFR-TKI could be interrupted, at least temporarily, when clinically warranted in patients with mRCC sufficiently controlled by TKI and duration of TKI therapy (< 1 year) before TKI discontinuation was an independent significant prognostic factor of poor PFS (*p* = 0.045) [21]. To date none of the cancer guidelines recommends sunitinib discontinuation for mRCC. Regarding our patient, it is remarkable that RCC relapsed after a disease-free period of more than 10 years, he presents an indolent disease managed by active surveillance before deliberately deferred sunitinib. He achieved a partial response with long-term disease stabilization response to sunitinib for 28 months. During a follow-up of 13 months, no signs of significant tumor growth could be found neither clinically nor in MRI performed; for this the discontinuation will be prolonged. Our observation and several smaller studies have suggested that discontinuation of antiangiogenic therapy is possible in carefully selected patients and may improve symptoms of toxicity without loss of response to the same targeted agent, which was usually restarted after relapse [22–24]. The selection of patient candidates for sunitinib discontinuation should be rational, taking into account favorable Heng prognostic risk criteria, long disease-free interval from the time of nephrectomy to the diagnosis of metastasis, indicates a biological pattern of slow growth, disease typically limited to one or two sites with good Eastern Cooperative Oncology Group Performance Status (ECOG PS), and have disease control for a long period under sunitinib for the benefit to be optimal as in our case. A strategy of periodic treatment breaks, therefore, may allow for a reduction in overall toxicity and increase in patient quality of life while maintaining overall disease control with these noncurative therapies.

## Conclusions

In summary, targeted agents have in general produced higher response rates, longer PFS or improved OS; their success is limited due to significant burden side effects and costs. However, possibly in those patients, in whom disease is stable for a longer time, either before or under treatment, if more than two dose reductions are required (total dose reduction > 25 mg), it is preferable to discontinue sunitinib therapy. Further data is required regarding the optimal duration of systemic therapy in exceptional responders to TKIs, and who among these responders will remain in disease control after discontinuation of therapy. Other investigations from larger cohort of patients is warranted before such an approach can be regarded as safe and research is needed to characterize the molecular determinants of response and resistance to targeted therapy.

## Abbreviations

CT: Computed tomography
Hb: Hemoglobin
IMDC: International Metastatic Renal Cell Carcinoma Database Consortium
mRCC: Metastatic renal cell carcinoma
MRI: Magnetic resonance imaging
OS: Overall survival
PFS: Progression-free survival
TKI: Tyrosine kinase inhibitor
VEGF: Vascular endothelial growth factor
VEGFR: Vascular endothelial growth factor receptor
